# Application of four dyes in gene expression analyses by microarrays

**DOI:** 10.1186/1471-2164-6-101

**Published:** 2005-07-25

**Authors:** Yvonne CM Staal, Marcel HM van Herwijnen, Frederik J van Schooten, Joost HM van Delft

**Affiliations:** 1 Department of Health Risk Analysis and Toxicology, Maastricht University, P.O. box 616, 6200 MD Maastricht, The Netherlands

## Abstract

**Background:**

DNA microarrays are widely used in gene expression analyses. To increase throughput and minimize costs without reducing gene expression data obtained, we investigated whether four mRNA samples can be analyzed simultaneously by applying four different fluorescent dyes.

**Results:**

Following tests for cross-talk of fluorescence signals, Alexa 488, Alexa 594, Cyanine 3 and Cyanine 5 were selected for hybridizations. For self-hybridizations, a single RNA sample was labelled with all dyes and hybridized on commercial cDNA arrays or on in-house spotted oligonucleotide arrays. Correlation coefficients for all combinations of dyes were above 0.9 on the cDNA array. On the oligonucleotide array they were above 0.8, except combinations with Alexa 488, which were approximately 0.5. Standard deviation of expression differences for replicate spots were similar on the cDNA array for all dye combinations, but on the oligonucleotide array combinations with Alexa 488 showed a higher variation.

**Conclusion:**

In conclusion, the four dyes can be used simultaneously for gene expression experiments on the tested cDNA array, but only three dyes can be used on the tested oligonucleotide array. This was confirmed by hybridizations of control with test samples, as all combinations returned similar numbers of differentially expressed genes with comparable effects on gene expression.

## Background

DNA microarray technology is widely used for gene expression analysis studies [1-5], as it is a high throughput technique by which the expression of all genes in a whole genome can be studied in a single assay. For many microarrays, the probe consists of cDNA or oligonucleotides spotted on a glass slide, and the target is fluorescent labelled cDNA (or cRNA). Both direct as well as indirect labelling protocols are applied: either, one target cDNA or cRNA is labelled with a single dye and hybridized on a microarray slide, or two targets are labelled with two different dyes, one for the reference and one for the test sample, and co-hybridized on a microarray slide. In dual label experiments, most often Cyanine 3 (Cy3) and Cyanine 5 (Cy5) are used as fluorescent dyes, although other dyes have been suggested [6]. In this way differential expression for thousands of genes between two different RNA samples can be measured simultaneously. Usually these experiments are time consuming, and, because microarray slides and fluorescent labels are expensive, the experiments are also high in costs. Moreover, several replicates need to be performed to increase statistical significance and to detect small differences in gene expression [7,8].

The application of four different dyes to label targets would be a major advantage as fewer microarrays will be required, leading to a reduction of costs and time without compromising gene expression data. A larger number of samples can be compared directly on a single microarray by labelling with more dyes, suggesting that fewer arrays will be required and that the hybridization design can be further optimized [9,10]. For instance, in the case that four samples need to be compared in all combinations, a dual-label common reference design requires four arrays for a single analysis of each sample, whereas a four-label design would require no common reference because all samples can be hybridized on a single array and only one array for a single analysis of each sample is needed. This will reduce variation, since variation between signal intensities for two dyes on a single spot is much smaller than variation between spots on different arrays [11]. Furthermore, day to day variation is reduced since it is possible to achieve more hybridizations on the same day [12]. In toxicogenomics assessments, as well as in other research areas, the approach to use multiple dyes can be of high value as it allows comparing several exposure conditions or time series simultaneously.

Forster et al [13] were the first to study the feasibility of using a third dye (Alexa 594) for labelling in microarray based gene expression analyses. Although they found that Alexa 594 gave a small signal in the Cy3 channel during scanning and Cy3 gave a small signal in the Alexa 594 channel, they concluded that Alexa 594 could be used besides Cy3 and Cy5 for direct comparison of two experimental samples and measuring these samples in relation to a reference sample.

The goal of our study was to investigate whether more than three different fluorescent dyes can be applied in gene expression studies using DNA microarrays. This was studied using microarrays with cDNA and oligonucleotide probes by hybridizing with a single sample labelled with four dyes (a quadruple self-hybridization or further stated as self-hybridization). Self-hybridization experiments are useful for measuring microarray data variability since any deviation from the expected value of 0 (for log transformed data) is caused by systemic or technical variation [13,14]. We also studied the application of more than two dyes for gene expression changes caused by exposure of cells to benzo[a]pyrene, to verify that the new dyes can be applied simultaneously in microarray studies. In the present study, we demonstrate that on our cDNA arrays four dyes can be applied, but that hybridization on the oligonucleotide arrays should be restricted to three dyes.

## Results

### Selection of fluorescent dyes

Four different dyes were tested for signal cross-talk at the emission / excitation settings of the ScanArrayExpress, namely Alexa 488, Alexa 594, Cyanine 3 and Cyanine 5. Therefore, the fluorescence of each dye at scanner settings of all tested dyes was measured. Results are summarized in Table 1. Since none of these dyes gives hardly any signal at settings for any other dye, it can be concluded that all dyes can be used simultaneously and were therefore considered suitable for use in microarray experiments. These dyes were further examined on two different microarray platforms.

**Table 1:** Cross-fluorescence of tested dyes. Fluorescence of dyes at scanner settings of all dyes expressed as a percentage of fluorescence at its own settings (the latter was set to 100%).

dye	Alexa 488	Alexa 594	Cy3	Cy5
settings				
Alexa 488	100.0	0.0	1.1	0.0
Alexa 594	0.9	100.0	1.3	13.0
Cy3	0.0	2.2	100.0	0.0
Cy5	0.1	0.1	0.2	100.0

### Optimizing laser power and PMT gain settings

**Figure 1:**
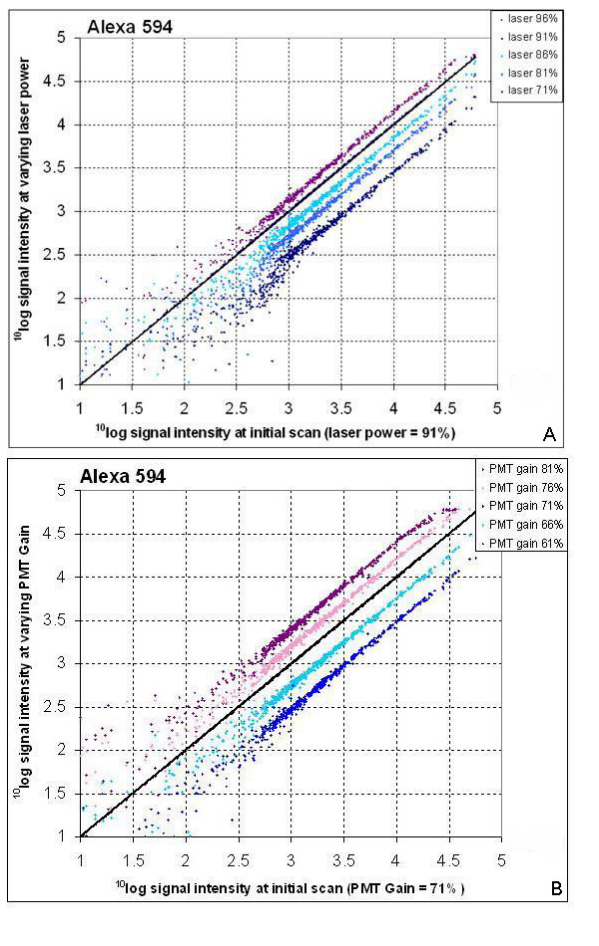
** (A) Effect of varying laser power settings on Alexa 594 fluorescence signals**. Results for the PHASE-I cDNA microarray scanned with constant PMT gain and varying laser power. Average ^10^log transformed fluorescence data for each gene of the dyes at varying setting (y-axis) was plotted against the initial fluorescence data (x-axis).** (B) Effect of varying PMT gain settings on Alexa 594 fluorescence signals**. Results for the PHASE-I cDNA microarray scanned with constant laser power and varying PMT gain. Average ^10^log transformed fluorescence data for each gene of the dyes at varying setting (y-axis) was plotted against the initial fluorescence data (x-axis)

The cDNA microarray from PHASE-I Molecular Toxicology was hybridized with a single cDNA target labelled with four different dyes (Cy3, Cy5, Alexa 488 and Alexa 594). Initial laser power settings for Alexa 488, Alexa 594, Cyanine 3 and Cyanine 5 were respectively 93, 91, 89 and 80%, and initial PMT gain settings were respectively 72, 71, 61, 60%. In order to obtain the optimal scan settings for each dye, the array is scanned at different laser power and PMT gain setting. Figure 1 shows, as an example, the data for varying laser power and PMT gain settings for Alexa 594.

In the scatter plots of data of one scan versus another, in general the data points indicate parallel lines when the settings are varied between the scans, implying that the fluorescent signals are consistent for all levels of gene expression when targets are labelled with these dyes. The larger distribution of the data points at low signals is a normal effect, which is due to reduced accuracy to measure signals from low expressed genes. Compared to Alexa 594, varying laser settings gave similar results for Alexa 488. For Cy3 and Cy5, the data points in the scatter plots run parallel for each setting. Varying laser settings gave similar results for all tested dyes. The Alexa 488 and Alexa 594 graphs, however, show a minor disturbance in the lines of the data points when the laser is varied (shown for Alexa 594 in Figure 1a). This suggests that for these two dyes, a fixed laser power should always be applied, whereas the other dyes allow some variation. Furthermore, these data indicate that laser power and PMT gain can be varied to some extend without affecting relative gene expression levels, as long as there is no saturation of signal intensities.

**Figure 2:**
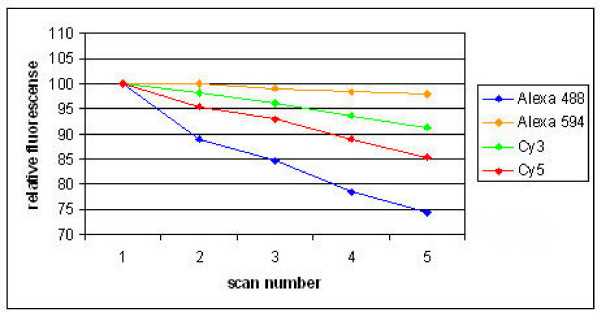
**Photo bleaching of Alexa 488, Alexa 594, Cy3 and Cy5 after repetitive scanning of the microarray**. Mean signal intensity of Alexa 488, Alexa 594, Cy3 and Cy5 is presented after repetitive scanning, relative to the signal at the first scan.

We also tested photo bleaching of the 4 dyes by scanning the microarray slide up to 5 times with the same scanner settings for all 4 dyes, and plotted the mean signal intensities as percentage of the signal intensity at the first scan (Figure 2). As is evident, photo bleaching occurs for all dyes as for all the signals decreases. The reduction was highest for Alexa 488 and least for Alexa 594, but was always small (<11% between the first and second round of scanning). Furthermore, the signal-to-noise ratio did not change for either of the dyes after repetitive scanning (data not shown). Therefore, we conclude that the photo bleaching is not expected to hamper gene expression analyses on microarrays.

### Correlation coefficients between dyes at different laser power settings

The influence of laser power and PMT gain settings on the correlations between the combinations of dyes to a trend line was examined. A cDNA microarray was scanned at the initial settings (mentioned above), and with a laser power of 70% or 100% and with adjusted PMT gain until none of the spots gave saturated signals. Results are shown in the Table 2. These correlation coefficients show that for all possible combinations of dyes, increasing the laser power, and thereby reducing the PMT gain, results in a higher correlation coefficient. This suggests that these cDNA microarrays with targets labelled with Alexa 488, Alexa 594, Cy3 and Cy5, and scanned with the ScanArrayExpress, could best be scanned at 100% laser power setting and adjusted PMT gain settings, in order to obtain the smallest variation in gene expression values. Although the correlations are high and differences are marginal, the poorest correlation for the first array was found for Alexa 488 combined with Cy5 (0.935), and the highest correlation for Alexa 594 with Cy3 (0.988).

**Table 2:** Correlation coefficients between gene expression measured for different dyes at various laser settings for the cDNA array. Correlation coefficients for all genes on the PHASE-I cDNA array between combinations of dyes at different scanner settings for 1 array, and at laser power 100 settings for 5 arrays.

	array1			laser power = 100 (n = 5)
	
Settings	laser = 70	original settings	laser = 100	mean ± stdev
Dye combination				
Alexa 488 vs Alexa 594	0.953	0.967	0.965	0.953 ± 0.020
Alexa 488 vs Alexa Cy3	0.916	0.941	0.955	0.923 ± 0.044
Alexa 488 vs Cy5	0.890	0.935	0.935	0.942 ± 0.014
Alexa 594 vs Cy3	0.958	0.983	0.988	0.938 ± 0.053
Alexa 594 vs Cy5	0.946	0.978	0.979	0.986* ± 0.005
Cy3 vs Cy5	0.975	0.981	0.987	0.926* ± 0.062

Mean	0.940	0.964	0.968	0.945 ± 0.025

Original scanner settings for Alexa 488, Alexa 594, Cy3 and Cy5 are: laser power 93, 91, 89 and 80% respectively, and PMT Gain 72, 71, 91, 60% respectively.

* The correlation coefficient between Alexa 594 – Cy5 is significantly higher than the correlation coefficient for any dye combination with Alexa 488, and the combination Alexa 594-Cy3 has a significantly higher correlation coefficient than Cy3-Cy5 (t-test, p < 0.05).

The reproducibility was tested by several other self-hybridizations of different RNA samples. Table 2 shows the results for the correlation coefficients calculated for all combinations of dyes. Numerical data from the table indicate that correlation coefficients for the repeated experiments are similar with mean correlation coefficients varying between 0.923 (Alexa 488 and Cy3) and 0.986 (Alexa 594 and Cy5).

For the rat oligonucleotide microarray, also self-hybridizations with targets labelled with Cy3, Cy5, Alexa 488 and Alexa 594 were also conducted and the laser power was set to 70 or 100% with adjustment of the PMT gain until no saturation of fluorescence occurred. Table 3 represents the correlation coefficients for these settings, and similar on this array, the correlations for all combinations of dyes are higher at laser power settings of 100% compared to 70%. However in all cases the correlation coefficients were smaller (varying between 0.486 and 0.887) compared to the cDNA array. Furthermore, Table 3 shows that correlations between Alexa 488 and any other dye are much lower than the correlation for any of the other combinations. This is probably due to the high background fluorescence for Alexa 488 on these arrays compared to the spot signals. The ratio of mean spot signal to mean background variation (signal-to-noise ratio) was clearly lower for Alexa 488 then for the other dyes (namely, 1.25, 1.65, 2.88 and 1.88 for Alexa 488, Alexa 594, Cy3 and Cy5, respectively). The high background signal in the Alexa 488 channel can not be due to auto-fluorescence of the Corning slides alone as it was not observed when scanning an unhybridized microarray.

**Table 3:** Correlation coefficients between gene expression measured for different dyes at various laser settings for the oligonucleotide array. Correlation coefficients for all genes on the oligonucleotide array between combinations of dyes at different scanner on 1 array, and at laser power 100 settings for 7 arrays. Alexa 488 labelled samples were only hybridized on the first array.

	array1		laser power = 100 (n = 7)
	
Settings	laser = 70	laser = 100	mean ± stdev
Dye combination			
Alexa 488 vs Alexa 594	0.118	0.561	
Alexa 488 vs Alexa Cy3	0.080	0.512	
Alexa 488 vs Cy5	0.127	0.486	
Alexa 594 vs Cy3	0.334	0.857	0.855 ± 0.032
Alexa 594 vs Cy5	0.279	0.808	0.853 ± 0.027
Cy3 vs Cy5	0.313	0.887	0.890* ± 0.052

Mean	0.208	0.685	0.843 ± 0.072

* The correlation coefficient between Cy3 – Cy5 is significantly higher than the correlation coefficient of Alexa 594 – Cy3 (t-test, p < 0.05).

To reduce the background binding on the oligonucleotide arrays, we applied several different hybridisation and washing protocols. We varied BSA concentration in the hybridization buffer, added tRNA, Cot1 or PolyA and used a commercial hybridization buffer (DIG Easy Hyb granules, Roche, Germany). We also varied the concentrations SSC and SDS in the washing buffers. The best results for all dyes were obtained by using the hybridization protocol as described in "Microarray hybridizations" of the Methods section. The data from this most optimal protocol are presented here.

With the exclusion of Alexa 488, the other dyes were tested in several more self-hybridizations with for each array a different RNA sample in order to confirm the reproducibility. Table 3 shows the correlation coefficients for all combinations of the 3 dyes. The correlation coefficients are similar for all repetitive experiments with mean values varying between 0.854 and 0.891.

### Standard deviation in relation to spot intensity for all combinations of dyes

The standard deviation for the ^10^log transformed expression ratios of the 3 or 4 replicate spots per gene on the arrays was calculated and plotted against the mean signal intensity of the corresponding dyes (Figure 4). For both arrays, the standard deviation decreased with increasing gene expression level. For the cDNA array, the standard deviation was equal for all combinations of dyes at a ^10^log signal intensity of 3 and higher. At lower signal intensities, however, the standard deviation for combinations of any dye with Alexa 488 were higher than for Cy3-Cy5 combinations, and standard deviations for combinations with Alexa 594 are intermediate. For the oligonucleotide array, the standard deviation for all combinations of dyes with Alexa 488 is higher at any signal intensity than for any other combination of dyes.

**Figure 3:**
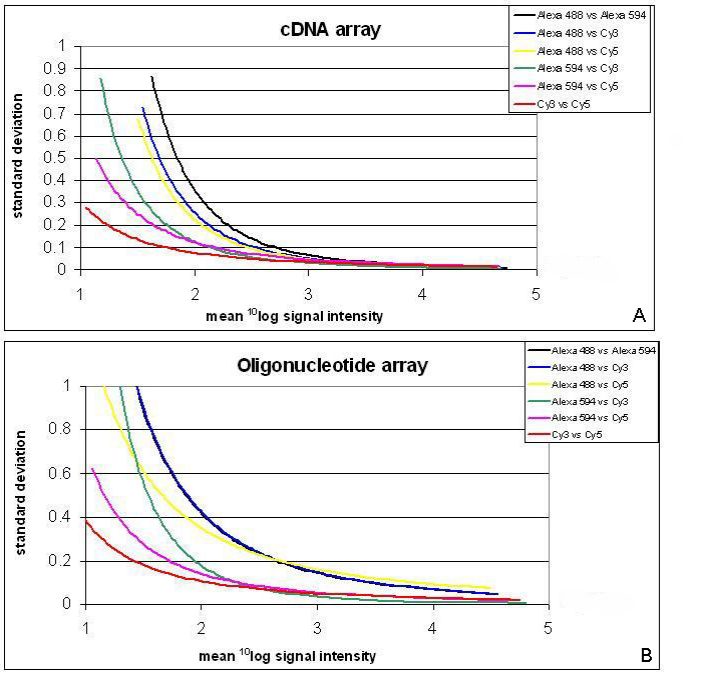
** (A) Standard deviation of the expression ratio to the relative expression level for the PHASE-I cDNA array**. Standard deviation of ^10^log transformed expression ratios for the 4 replicate spots of each gene (y-axis) plotted against the mean ^10^log transformed signal intensities (x-axis) for the corresponding dyes for all combinations of dyes and for the cDNA array. Regression lines are based on a power model.** (B) Standard deviation of the expression ratio to the relative expression level for the Oligonucleotide array**. Standard deviation of ^10^log transformed expression ratios for the 3 replicate spots of each gene (y-axis) plotted against the mean ^10^log transformed signal intensities (x-axis) for the corresponding dyes for all combinations of dyes and for the oligonucleotide array. Regression lines are based on a power model.

### Identification of modulated genes for various dye combinations

As microarrays are intended to identify genes that are differentially expressed between different RNA samples, we tested the applicability of four dyes by analyzing RNA samples from cells exposed to 3 concentrations of B[a]P versus a vehicle control. Table 4 shows the labelling and hybridization schedule for the B[a]P exposed samples on the arrays (per array, four RNA samples were simultaneously hybridized), which was conducted to the two independent treatments (see Materials and Methods). Every dye was used for every RNA sample, but not each dye combination was applied for each combination of control and test sample. For every B[a]P concentration a confidence analysis was performed to select modulated genes for each dye combination separately. Also, for all dye combinations combined (paired data), a confidence analysis was conducted. For the cDNA array 20, 31 and 45 genes were found modulated for paired data of respectively 3, 10 and 30 μM. For the oligonucleotide array 121, 97 and 195 genes were found modulated for paired data of respectively 3, 10 and 30 μM. Modulated genes for each dye combination were compared to modulated genes found all dye combinations paired. Table 5 and 6 summarize the results for respectively the cDNA arrays and the oligonucleotide arrays; they present numbers of modulated genes for specific dye combinations as a percentage of numbers of modulated genes by all dye combinations combined (in bold). On average, this percentage is approximately 45%, although in some cases it is clearly lower or higher. This deviation, however, is not consistent for a dye or a combination of dyes, so it can be concluded that all dyes perform equally well in identifying differentially expressed genes. Also in these Tables, the different dye-combinations are compared to each other, all as a percentage of modulated genes by all dye combinations (in italics). Once again differences are observed, which are not sufficient consistent to conclude that one combination of dyes performs worse or better than another to identify modulated genes.

**Table 4:** Labelling schedule for B[a]P exposed samples. Each HepG2 sample is labelled with each fluorescent dye. Rat liver samples were labelled as shown by array number 1–3, without the application of Alexa 488.

fluorescent label array no.	Cy3	Cy5	Alexa 594	Alexa 488
1	0 μM	3 μM	10 μM	30 μM
2	10 μM	0 μM	30 μM	3 μM
3	3 μM	30 μM	0 μM	10 μM
4	30 μM	10 μM	3 μM	0 μM

**Table 5a:** Performance of a dye combination in revealing modulated genes in B[a]P (3 μM) treated HepG2 cells using a cDNA array.

	Cy5-Cy3	Cy3-A594	A488-Cy5	A594-A488
Cy5-Cy3	50*			
Cy3-A594	*40*	**50**		
A488-Cy5	*25*	*35*	**50**	
A594-A488	*40*	*40*	*25*	**40**

* In bold the intersection of two gene lists indicating the modulated genes for a dye combination as percentage of all modulated genes (20 genes) found by analysis of all dye combinations combined. In italics the intersection of three gene lists indicating the modulated genes for two dye combinations as percentage of all modulated genes found for all dye combinations combined. The first dye was used for B[a]P treatment, the second for the control.

**Table 5b:** Performance of a dye combination in revealing modulated genes in B[a]P (10 μM) treated HepG2 cells using a cDNA array.

	Cy3-Cy5	A594-Cy3	Cy5-A488	A488-A594
Cy3-Cy5	29*			
A594-Cy3	*23*	**58**		
Cy5-A488	*6*	*10*	**16**	
A488-A594	*26*	*39*	*13*	**74**

* In bold the intersection of two gene lists indicating the modulated genes for a dye combination as percentage of all modulated genes (31 genes) found by analysis of all dye combinations combined. In italics the intersection of three gene lists indicating the modulated genes for two dye combinations as percentage of all modulated genes found for all dye combinations combined. The first dye was used for B[a]P treatment, the second for the control.

**Table 5c:** Performance of a dye combination in revealing modulated genes in B[a]P (30 μM) treated HepG2 cells using a cDNA array.

	Cy3-A488	A488-Cy3	Cy5-A594	A594-Cy5
Cy3-A488	35*			
A488-Cy3	*24*	**53**		
Cy5-A594	*22*	*33*	**38**	
A594-Cy5	*16*	*20*	*16*	**31**

* In bold the intersection of two gene lists indicating the modulated genes for a dye combination as percentage of all modulated genes (45 genes) found by analysis of all dye combinations combined. In italics the intersection of three gene lists indicating the modulated genes for two dye combinations as percentage of all modulated genes found for all dye combinations combined. The first dye was used for B[a]P treatment, the second for the control.

**Table 6:** Performance of a dye combination in revealing modulated genes in B[a]P treated liver slices using an oligonucleotide array.

B[a]P concentration dye combination	3 μM		10 μM		30 μM	
Cy5 – Cy3	36*	*11*				
Cy3 – A594	**64**					
Cy3 – Cy5			**47**	*13*		
A594 – Cy3			**48**			
Cy5 – A594					**51**	*8*
A594 – Cy5					**38**	

* In bold the intersection of two gene lists indicating the modulated genes for a dye combination as percentage of all modulated genes (for 3, 10 and 30 μM: 121, 97 and 195 genes respectively) found by analysis of all dye combinations combined. In italics the intersection of three gene lists indicating the modulated genes for two dye combinations as percentage of all modulated genes found for all dye combinations combined. The first dye was used for B[a]P treatment, the second for the control.

**Figure 4:**
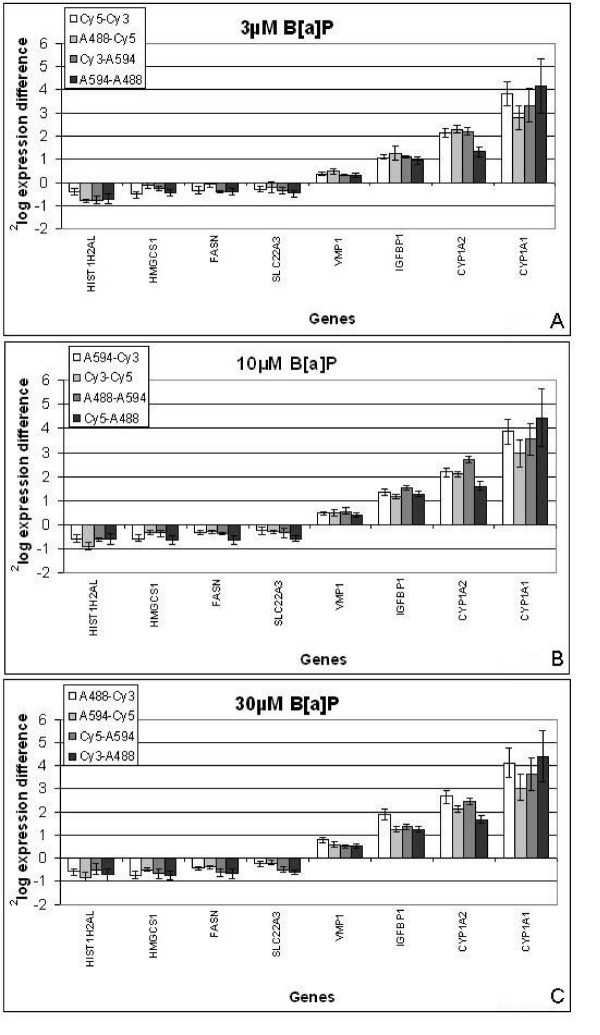
** (A) Gene expression difference for several genes after exposure of HepG2 cells to 3 μM B[a]P during 6 h**. Gene expression difference for several genes, in varying relative gene expression level of high (FASN and HIST1H2AL), middle (CYP1A2, HMGCS1, VMP1 and IGFBP1) and low (CYP1A1, SLC22A3), as measured by different dye combinations by using four dyes simultaneously on cDNA microarrays in RNA samples from HepG2 cells exposed to 3 μM B[a]P during 6 hours. Error bars indicate the standard deviation for the replicate spots.** (B) Gene expression difference for several genes after exposure of HepG2 cells to 10 μM B[a]P during 6 h**. Gene expression difference for several genes, in varying relative gene expression level of high (FASN and HIST1H2AL), middle (CYP1A2, HMGCS1, VMP1 and IGFBP1) and low (CYP1A1, SLC22A3), as measured by different dye combinations by using four dyes simultaneously on cDNA microarrays in RNA samples from HepG2 cells exposed to 10 μM B[a]P during 6 hours. Error bars indicate the standard deviation for the replicate spots. ** (C) Gene expression difference for several genes after exposure of HepG2 cells to 30 μM B[a]P during 6 h**. Gene expression difference for several genes, in varying relative gene expression level of high (FASN and HIST1H2AL), middle (CYP1A2, HMGCS1, VMP1 and IGFBP1) and low (CYP1A1, SLC22A3), as measured by different dye combinations by using four dyes simultaneously on cDNA microarrays in RNA samples from HepG2 cells exposed to 30 μM B[a]P during 6 hours. Error bars indicate the standard deviation for the replicate spots.

Additionally, the performance of the dye combinations was evaluated by comparing the gene expression difference. Figure 4, which represents the results for the experiment with HepG2 cells on DNA microarrays with the application of four dyes simultaneously, can be used as an example. For each dye combination a similar effect on gene expression is observed and it can be summarized that all dye combinations result in similar gene expression changes. For the rat liver slices similar results were found.

## Discussion

We have investigated the applicability of four fluorescent dyes in gene-expression analysis by microarrays. By using more than two dyes in microarray experiments, without lessening the data obtained, costs and time can be decreased as fewer microarrays are needed.

Initially, several dyes were tested for cross-talk on the ScanArrayExpress reader, and ultimately 4 dyes were tested for parallel use in microarray experiments. Today, Cy3 and Cy5 are the most widely used dyes in microarray experiments and much research has been done on these dyes [4,8,11,15,16], although Alexa 555 and Alexa 647 have been suggested by Cox et al [6]. It was our intention to select dyes that could complement Cy3 and Cy5 and we show that Alexa 488 and Alexa 594 are suited for this and can be used for parallel hybridization in microarray experiments. All dyes were applicable on the tested cDNA arrays. On the tested oligonucleotide arrays, however, only three dyes, namely Alexa 594, Cy3 and Cy5, could be used.

### Selection of fluorescent dyes

Based on cross-talk signals, four dyes – Alexa 488, Alexa 594, Cy3 and Cy5 – were found suitable for hybridization on microarrays and some cross-talk did occur for this combination. The highest fluorescence for a dye at settings of another dye was observed for Cy5, namely 13% cross-talk at the settings for Alexa 594. This cross-talk may influence differential gene expression analyses, especially if the signals for Cy5 and Alexa 594 differ drastically within a spot. Therefore, in order to minimize artificial gene expression differences, scan settings should be optimized such that emission intensities are gross similar (e.g. by assuring that the brightest spots are on the edge of saturation). Furthermore, dye swap design on replicate arrays will reduce the bias resulting from cross-talk, and algorithms can be developed to eliminate this bias.

### Dye bias

Dye bias is the difference in labelling efficiency between different dyes as one dye can be better incorporated than another; this can affect the gene expression data [17-19]. When using more than one dye, dye bias may occur and most likely, it is enhances with increasing number of dyes. Dye bias can be reduced by using the indirect amino-allyl labelling instead of direct labelling, but it is not clear whether dye bias is fully eliminated [11]. However, dye bias can be eliminated by LOWESS normalization of the data, combined with a labelling and hybridization design in which each target is labelled with each different dye [20]. Liang et al [7] showed that the correlation between predicted and observed gene expression ratios increased by adding a second microarray with dye switching. This confirms that accuracy can be improved by adding dye swap replicates and applying a balanced labelling design. A balanced labelling design with four dyes may increase the number of required arrays, but still saves the total number of arrays. For example, when 3 treatments and a control are to be compared using 4 data points per comparison, 16 microarrays are needed for a common reference design, 12 arrays for a block design (treatment vs. control on an array), 8 when using a loop design, but only 4 with 4 dyes and the design shown in Table 4.

### Applicability of selected dyes

The applicability of the dyes was analyzed in four different ways. First by calculating the correlation coefficients between dyes in self-hybridizations, second by calculating the standard deviation of their log ratio per gene for replicate spots in the self-hybridizations, third by comparing numbers of modulated genes for all dye combinations in samples exposed to B[a]P and finally by comparing gene expression modulation for several genes from samples exposed to B[a]P.

When applied on the cDNA array, all combinations of dyes gave high correlation coefficients (>0.9) and thus seem suitable for parallel hybridization in microarray experiments. On the oligonucleotide array, the correlation coefficients were high for all combinations (>0.8), except for combinations with Alexa 488 (≌0.5). The correlation coefficients for all combinations of dyes on both arrays are constant in multiple repeated hybridizations. These results are supported by the plots for the standard deviation of the replicate spots. For the cDNA array, the standard deviation is equal for all combinations of dyes at high gene expression level. However, for the oligonucleotide array the standard deviation of the signal intensity of high expressed genes for all combinations with Alexa 488 is higher than the standard deviation for all other combinations of dyes. Since the correlation coefficient of Alexa 488 with other dyes is low and the standard deviation for Alexa 488 is high, it is not advisable to use Alexa 488 for labelling and hybridization on the oligonucleotide array.

The correlation coefficients observed for all combinations of Alexa 488 with any other dye on the oligonucleotide array are lower than any of the other correlation coefficients. This was due to a high background signal and a lower signal-to-noise ratio in the Alexa 488 channel, which can not be attributed to auto fluorescence. This background signal was much less pronounced on the cDNA array, which may be explained by a different coating of the microarray slides. Alexa dyes have a net negative charge, which may cause non-specific electrostatic interaction with positively charged molecules [21]. This may be a reason for why the dye adhered differently to the two different microarray slides. However, this does not explain why the background binding for Alexa 594 is much less in comparison to that of Alexa 488.

For all dyes tested on the oligonucleotide array, many genes showed a low gene expression level compared to the cDNA array. In general, weak signals are detected with lower accuracy than strong ones [22]. This is reflected by the higher standard deviations for lower signals in the plots for the cDNA and oligonucleotide array (Figure 4). Lyng et al [22] showed that reliable data for mean signal intensities were only achieved within a range of 200 to 50,000 (no background correction performed). This clarifies the lower correlation coefficients found for the oligonucleotide array compared to the cDNA array.

For all dye combinations, percentages of modulated genes relative to modulated genes for all dye combinations combined are generally equal (Tables 5 and 6). This indicates that any dye combination has approximately the same sensitivity to identify differentially expressed genes, and that the traditional combination of Cy3-Cy5 is not necessarily preferable above the others. Therefore, we consider all dyes suitable for usage in gene expression studies by microarrays. This was further substantiated by the observation for several differentially expressed genes that the level of modulation is in the same range for all dye combinations.

Although Forster et al [13] used a different approach to test the use of Alexa 594 besides Cy3 and Cy5 in microarray analysis, their conclusions are in agreement with that of this study. Forster et al [13] tested the use of different combinations of two dyes in hybridization, and found some cross-talk between Cy3 and Alexa 594 and between Cy5 and Alexa 594. Although, some cross-talk was observed between Cy5 and Alexa 594 (13%), only small cross-talk was noticed (<3%) for Cy3 and Alexa 594 in this study. Forster et al [13] also found a more linear relation between Cy3 and Alexa 594 than for Cy3 and Cy5. However, we noticed only a small difference in correlation coefficient for Cy3 / Cy5 and for Alexa 594 / Cy3 (Table 2 and 3). These differences could be due to the different testing methods and different arrays used.

## Conclusion

All our experiments demonstrate that for gene expression analyses on microarrays Alexa 594 is best suited as a third dye in addition to Cy3 and Cy5, and that Alexa 488 can be applied as a fourth dye on some microarray platforms, but unfortunately not on all array platforms. The general applicability of four dyes on other microarray systems is therefore uncertain, and needs to be investigated on a case-by-case basis.

## Methods

### Cross-talk analysis of fluorescent dyes

Two ARES™ Alexa fluor^® ^dyes (Alexa 488 and 594) (Molecular Probes, Leiden, The Netherlands) and conventionally used Cyanine3 (Cy3) and Cyanine5 (Cy5) (Amersham Biosciences, Uppsala, Sweden) were tested for cross-talk of excitation / emission signals. All dyes were dissolved according to the producer's manual and applied on a glass slide. The slide was scanned with a ScanArrayExpress microarray scanner (Packard BioChip Technologies, Perkin Elmer life sciences, Boston, USA) with laser wavelengths for Alexa 488, Cy3, Alexa 594 and of 488, 543.8, 594 and 632.8 nm respectively, and emission filter of 522, 570, 614 and 670 nm respectively. The images were analyzed with ImaGene (BioDiscovery, USA). Fluorescence of each dye at the scanning settings of all tested dyes was measured and four dyes were selected for further use (see Results).

### Source of RNA samples

RNA was isolated from cultured HepG2 cells or from rat liver or precision-cut liver slices and used for the microarray hybridizations. HepG2 cells were cultured in Minimal Essential Medium (MEM) supplemented with 1% non-essential amino acids, 1% sodium-pyruvate, 2% penicillin/streptomycin and 10% Foetal Bovine Serum (all from Gibco/BRL, Breda, The Netherlands) in T25 culture flasks at 37°C and 5% CO2. HepG2 cells were exposed to 3, 10 or 30 μM of Benzo[a]pyrene (B[a]P, from Sigma-Aldrich, the Netherlands) and a vehicle control (DMSO) during 6 hours in two independent experiments. DMSO concentration in de cell culture media was 0.1%. After exposure, media was removed and 1 ml Trizol (Gibco/BRL, Breda, The Netherlands) was immediately added to the cells.

A male Wistar albino rat (200 g) was killed by cervical dislocation, and the liver after removal, was snap frozen in liquid nitrogen and stored at -80°C. Liver tissue (8.6 g) was crushed using a mortar and pester. An amount of 0.05 g crushed liver tissue was dissolved in 1 ml Trizol reagent. Additionally, precision-cut liver slices were obtained by using a Krumdieck tissue slicer [23]. Cylindrical liver cores with a diameter of 8 mm were sliced into 250 μm thick slices. In the two independent experiments, slices were exposed to 3, 10 or 30 μM B[a]P or a solvent control (DMSO 0.067%) during 24 hours. After exposure, slices were snap frozen in liquid nitrogen and RNA was isolated in a manner similar to that of the whole liver tissue.

### RNA isolation and cDNA synthesis

RNA was isolated from the Trizol solutions according to the producer's manual and purified with the RNeasy mini kit (Qiagen Westburg bv., Leusden, The Netherlands). RNA quantity was measured on a spectrophotometer and the quality was determined on a BioAnalyzer (Agilent Technologies, Breda, The Netherlands). Only RNA samples which showed clear 18S and 28S peaks were used for labelling and hybridization. In order to generate sufficient large uniform samples for the multiple self hybridizations, RNA samples from several isolations were pooled.

RNA was reverse transcribed into cDNA in quadruplicate with amino allyl labelled dUTP (Sigma-Aldrich, St Louis, USA) and subsequently labelled with one of the dyes (based on Van Delft et al [24]). For each sample, a mixture of 10 μg of RNA and 6 μg of random hexamer primers were incubated in 18.5 μl at 70°C for 10 minutes and snap frozen on dry ice / ethanol for 30 seconds. Thereafter DTT (final concentration 10 mM), 0.5 mM dATP, dCTP and dGTP, 0.3 mM dTTP, 0.2 mM 5-(3-aminoallyl)-2'deoxyuridine-5'-triphosphate (aa-dUTP), and 400 U Superscript II reverse transcriptase (Invitrogen, Life Technologies, Breda, The Netherlands) were added to a final volume of 30.1 μl and incubated overnight at 42°C. RNA was hydrolyzed by adding 10 μl of 1 M NaOH and 10 μl of 0.5 M EDTA followed by an incubation of 15 minutes at 65°C. To neutralize, 10 μl of 1 M HCl was added. cDNA samples were purified to remove unincorporated amino allyl dUTP and buffers using a QIAquick PCR Purification Kit (Qiagen Westburg bv., Leusden, The Netherlands) according to the producer's manual. However, in order to eliminate interference of amines during labelling, buffers were substituted by phosphate buffers (wash buffer: 5 mM KPO4 pH 8.0, 80% ethanol; elution buffer: 4 mM KPO_4 _pH 8.5). The sample was eluted in duplicate using 30 μl elution buffer and dried in vacuo. Following amino-allyl labelling, cDNA targets were resolved in 4.5 μl of 0.1 M Na_2_CO_3_ pH 9.0 and 4.5 μl of a 2.25 μM of Cy™5 or Cy™3 Monofunctional Reactive Dye esters (Amersham Biosciences, Uppsala, Sweden) was added. Samples were incubated in the dark at room temperature for 1 hour. Targets to be labelled with Alexa dyes were resolved in 5 μl of MilliQ, 3 μl of labelling buffer (Sodium bicarbonate, prepared according to the producers' manual) and 2 μl of a 6.3 μM of ARES™ Alexa Fluor^® ^(Molecular Probes, Leiden, The Netherlands) (dissolved in DMSO according to the producers' manual) was added. The sample was incubated for 1 hour in the dark at room temperature. After incubation 35 μl of 100 mM NaAc pH 5.2 was added to the Cy-labelled targets and 90 μl MilliQ was added to the Alexa labelled targets. The samples were purified using a QIAquick PCR Purification Kit (Qiagen Westburg bv, Leusden, The Netherlands) to remove unincorporated dyes. To the rat liver targets, additional 4 μl of 100 U/ml Poly-dA (Amersham Biosciences, Uppsala, Sweden) and 3 μl of 1 mg/ml mouse Cot1-DNA (Invitrogen, Breda, The Netherlands) were added to block an unspecific binding of the targets to the array. The targets were dried *in vacuo*.

### Microarray hybridizations

HepG2 targets were hybridized to the PHASE-1 Microarray Human-600 (PHASE-1 Molecular Toxicology, Santa Fe, USA), containing 597 sequence verified cDNA clones from human genes, representing a number of toxicologically relevant, as well as control, genes, each printed in quadruplicate. Hybridization and washing was done according to the producer's manual as previously described [15]. The labelled cDNA target was dissolved in 30 μl hybridization buffer (50% formamide, 5× SSC, 0.1% SDS, 0.1 mg/ml Salmon Sperm DNA) and incubated for 15 minutes in the dark at room temperature. The target was denatured by heating for 5 minutes at 95°C, centrifuged for 3 minutes at maximum speed, and placed in a heat block at 70°C until further use. The target (28 μl) was applied on the cover slip (24–32 mm) and the microarray was placed on top of the cover slip. The slide was hybridized overnight in a humidified hybridization chamber (Corning, Life Sciences, The Netherlands) in a water bath at 42°C. After incubation, the slide was placed in wash buffer (2× SSC, 30–34°C) to remove the cover slip, and washed 5 minutes in 2× SSC / 0.1% SDS, 5 minutes in 0.1× SSC / 0.1% SDS, 2 times 5 minutes in 0.1× SSC at 32°C, and 1 minute in MilliQ all at room temperature. The slide was centrifuged to dry.

Rat liver targets were hybridized on an Operon rat oligonucleotide array containing 5700 oligonucleotides (Operon, Qiagen, Venlo, The Netherlands) printed in triplicate on Corning UltraGAPS Coated Slides (Corning Life Sciences, New York, USA) by the Genome Centre Maastricht (Maastricht University, Maastricht, The Netherlands). Hybridization and washing was done according to Corning's protocol for oligonucleotide arrays. The labelled cDNA target was dissolved in 65 μl hybridization buffer (30% formamide; 5× SSC; 0.1% SDS) and incubated for 15 minutes in the dark at room temperature. The target was denatured by heating for 5 minutes at 95°C, centrifuged for 2 minutes at maximum speed, and kept at room temperature until further use. The microarray slide and cover slip (24 × 60 mm) were prehybridized for 45 minutes in preheated prehybridization buffer (5× SSC; 0.1% SDS; 1% BSA) at 42°C. Slides and cover slips were washed several times in MilliQ followed by dipping in isopropanol and centrifugation to dry. The target (60 μl) was applied on the cover slip and the microarray was placed on top of the cover slip. The slide was hybridized overnight in a humidified hybridization chamber (Corning, Life Sciences, The Netherlands) in a water bath at 42°C. After incubation, the slide was placed in wash buffer (2× SSC / 0.1% SDS) at 42°C to remove the cover slip. The slide was washed for 2 times 5 minutes in 2× SSC / 0,1% SDS at 42°C, 2 times 10 minutes in 0.1× SSC / 0.1% SDS at room temperature and 4 times 1 minute in 0.1× SSC at room temperature. The slide was centrifuged to dryness.

### Microarray data analysis

The microarray slides were scanned on a ScanArrayExpress (Packard Biochip Technologies, Perkin Elmer life sciences, Boston, USA). All four channels were scanned at several different settings for laser power and / or photo multiplier tube (PMT Gain). Settings were optimized such that the signal of the highest fluorescent spots is just below the maximum measurable level. Laser power settings were set at 100% and PMT Gain was adjusted, unless otherwise stated. The images (10 micron resolution; 16 bit tiff) were processed with ImaGene 5.0 software (BioDiscovery Inc., Los Angeles, USA) to quantify spot signals. Irregular spots were manually or automatically flagged and not included in the data analysis.

For the self-hybridizations, data from ImaGene were exported to Microsoft Excel (Microsoft, USA) for transformations and analysis. For each spot, mean local background signal was subtracted from the mean spot signal, negative signals were excluded, and the resulting net spot signal data were log transformed. These log transformed background corrected expression signals for all combinations of dyes at all scanner settings were plotted and analyzed by linear regression and correlation coefficients (R^2^) were calculated. Furthermore, standard deviations of ^10^log transformed expression ratios for each gene (for 3 or 4 replicate spots, depending on the array used), were plotted against the mean ^10^log transformed expression signals and analyzed by regression analysis.

For the B[a]P exposed samples, data from ImaGene were transported to GeneSight software version 4.1.5 (BioDiscovery Inc, Los Angeles, USA) for transformations and analyses. For each spot, background was subtracted; flagged spots and spots with a net expression level below 5 were omitted. Data were log base 2 transformed and expression difference between exposed and control were calculated. Data normalization was done by LOWESS and centring expression differences by subtracting mean values (the latter only for the oligonucleotide arrays). Data of replicate spots were combined while omitting outliers (>2 standard deviations). In order to estimate the number of differentially expressed genes following a treatment, the confidence analysis tool from GeneSight was used. For confidence analyses, for each B[a]P concentration, data of the two replicate arrays with the same dye combination were combined. Up-regulated and down-regulated genes were identified at 99% confidence intervals with up-regulation or down regulation levels set at 0.2 (^2^log-scale) for the cDNA arrays and respectively 99.5% and 0.5 for the oligonucleotide arrays.

## Authors' contributions

YS carried out the cell culturing and preparation of the liver slices including exposure and RNA isolation. YS and MvH carried out the labeling and hybridization of the samples and the image quantification. YS carried out the data analysis and drafted the manuscript. FvS and JvD participated in design of the study and preparation of the manuscript. All authors have read and approved the manuscript.
